# The legacy of larval infection on immunological dynamics over metamorphosis

**DOI:** 10.1098/rstb.2019.0066

**Published:** 2019-08-26

**Authors:** Justin T. Critchlow, Adriana Norris, Ann T. Tate

**Affiliations:** Department of Biological Sciences, Vanderbilt University, Nashville, TN, USA

**Keywords:** immune system evolution, adaptive decoupling hypothesis, antimicrobial peptides, ontogeny, gregarines, early-life exposure

## Abstract

Insect metamorphosis promotes the exploration of different ecological niches, as well as exposure to different parasites, across life stages. Adaptation should favour immune responses that are tailored to specific microbial threats, with the potential for metamorphosis to decouple the underlying genetic or physiological basis of immune responses in each stage. However, we do not have a good understanding of how early-life exposure to parasites influences immune responses in subsequent life stages. Is there a developmental legacy of larval infection in holometabolous insect hosts? To address this question, we exposed flour beetle (*Tribolium castaneum*) larvae to a protozoan parasite that inhabits the midgut of larvae and adults despite clearance during metamorphosis. We quantified the expression of relevant immune genes in the gut and whole body of exposed and unexposed individuals during the larval, pupal and adult stages. Our results suggest that parasite exposure induces the differential expression of several immune genes in the larval stage that persist into subsequent stages. We also demonstrate that immune gene expression covariance is partially decoupled among tissues and life stages. These results suggest that larval infection can leave a lasting imprint on immune phenotypes, with implications for the evolution of metamorphosis and immune systems.

This article is part of the theme issue ‘The evolution of complete metamorphosis'.

## 1. Introduction

Few factors have a greater impact on the outcome of an interaction between host and parasite, or the spread of disease in a host population, than the age and stage of the host. As hosts age, cumulative exposure to microbes shapes the maturation and polarization of their immune systems. Life-history priorities shift from growth to reproduction, inducing alterations in behaviour, food source and even ecological niche [1]. As a result, hosts experience dynamic changes over their ontogeny, from birth to old age, in both exposure to parasites and susceptibility to infection once exposed [2].

The consequences of these ontogenetic dynamics can be observed across broad swaths of the tree of life. In plants, for example, gibberellin hormones that promote seedling growth also inhibit signals related to defence against predators and parasites. At the same time, signalling from the salicylic acid pathway, which is involved in the response to biotrophic pathogens, inhibits the action of the growth hormones [3]. As a result, growing plants are susceptible to different pathogens than mature stages, and infection can influence the growth trajectories of their plant hosts [4]. In humans, lack of early-life exposure to beneficial microbes and other environmental antigens can set the stage for chronic inflammation, allergy and other forms of immunopathology [5,6]. From an evolutionary perspective, the risk of immunopathology in early life is predicted to favour decreased immunological sensitivity to infection later in life [7]. In all of these examples, host ontogeny is a fairly continuous process, punctuated by hormonal signals that encourage flowering or the onset of puberty but otherwise keep major organs and physiological structures intact. In animals that undergo metamorphosis, however, developmental continuity is swapped for discrete stages characterized by transition periods of dramatic physiological restructuring that alter the calculus of costs and trade-offs in host–parasite interactions.

During metamorphosis, tadpoles become frogs and caterpillars become butterflies, allowing hosts to exploit disparate resources and environments that individually maximize particular, stage-associated traits like growth or reproduction [8]. Metamorphosis is not a requirement for stage-specific niche differentiation; even within insects, dragonflies and mosquitoes both have an aquatic juvenile stage and a terrestrial adult stage, but dragonflies undergo relatively continuous maturation from instar to instar while the holometabolous mosquitoes undergo pupation prior to adulthood. Why bother with complete metamorphosis, then? After all, the pupal stage can be a liability as it is generally sessile, poorly defended and unable to acquire resources to fuel its energetic needs. The adaptive decoupling hypothesis suggests that the pupal stage might be the price paid for immature and mature developmental modules that can respond relatively independently to evolutionary pressures at a genetic regulatory level [9], allowing organisms to simultaneously maximize performance in multiple life stages.

The re-invention of the midgut during complete metamorphosis is a particularly potent example of adaptive flexibility achieved by decoupling one life stage from another. In most insects, the midgut comprises epithelial cells, goblet cells and stem cells [10–12]. The ratio and renewal rates of these cell types differ extensively from one life stage to another and vary dynamically even within life stages. For example, as larvae grow larger and moult, the stem cells of the midgut undergo proliferation and differentiate into new, polyploid epithelial cells and goblet cells [11]. This renewal process is also crucial in the host response to bacterial toxins and viruses that rely on the invasion of epithelial cells to colonize the host [13]. As insects transition to the pupal stage, however, the old somatic cells are excised into the lumen to form the yellow body, which undergoes apoptosis and autophagy to recycle the nutrients before being evacuated during eclosion of the new adult [12]. In the midgut of a new pupa, only the intestinal stem cells remain, imaginal structures that proliferate and differentiate into the epithelial cells that will eventually compose the adult gut [11]. Consistent with the adaptive decoupling hypothesis, the relative morphologies of larval and adult epithelial cells reflect the relative feeding ecologies of each life stage. For example, in fruit flies, the polyploid epithelial cells of larvae facilitate the rapid acquisition and processing of nutrients from complex food media, while the smaller, diploid nuclei of adult midgut epithelial cells reflect the narrower breadth of adult food sources [10]. On the other hand, the larval and adult stages of the flour beetle *Tribolium castaneum* both feed on the same resource [11], and both contain midgut epithelial cells that share a common polyploid morphology.

Midgut remodelling during insect metamorphosis can exert complex effects on the persistence of parasites and other microbes. Protozoan trophozoites that remain embedded in the flour beetle (*T. confusum*) gut when a larva enters metamorphosis, for example, are evacuated with the yellow body [14], allowing the adult to eclose without a parasite burden. On the other hand, the elimination of the gut epithelia could also eliminate beneficial microbiota, allowing any remaining opportunistic pathogens to exploit the pupa or colonize the new adult gut. Indeed *Galleria mellonella* moth pupae cooperate with a beneficial microbe (*Enterococcus mundtii*) to exclude pathogenic *Serratia* strains during metamorphosis [15]. Knocking down host immune gene expression or preventing the *E. mundtii* strain from producing bacteriocins allowed *Serratia* to dominate, at a cost to pupal survival. Furthermore, the cessation of resource acquisition during pupal gut remodelling can render larvally acquired infections hazardous during metamorphosis. The microsporidian parasite *Nosema whiteii* kills its flour beetle host during the pupal stage after manipulating the host into an extended larval stage during which the parasite converts acquired resources into spores [16]. Conversely, a protozoan parasite (*Ophryocystis elektroscirrha*) of the monarch butterfly (*Danaus plexippus*) can lethally deform its host during the pupal stage if it reaches excessive spore densities in the larval stage, prematurely curtailing transmission [17]. Thus, metamorphosis can shape parasite life-history evolution while also influencing host phenotypes and fitness.

The impact of metamorphosis on the adaptive decoupling of gene expression is hypothesized to extend to the immune system [18,19]. Life stages that use different resources or display disparate behaviours are also likely to encounter different types of parasites that require alternate forms of immunological defence. Thus, the decoupling of correlated gene expression by the use of different regulatory elements from one life stage to the next could allow evolution to simultaneously optimize immune system regulation in multiple life stages. Empirical evidence from multiple holometabolous insect species supports this hypothesis, as summarized in table 1. For example, the larvae and adults of *Drosophila melanogaster* fruit flies express the antimicrobial peptide diptericin at similar levels but fundamentally differ in their expression of the antimicrobial peptide drosomycin [19]. In a similar vein, the larvae of the *Anopheles gambiae* mosquito, which live in a microbe-rich aquatic environment, exhibit higher numbers of haemocytes that phagocytose bacteria and higher levels of immune gene expression than adults [18]. These examples suggest that expression of an immune phenotype in the larval stage does not indelibly predict adult phenotypes, allowing plasticity in immunological investment over ontogeny.

**Table 1.** The interaction of metamorphosis and immune function across holometabolous insect orders. AMP, antimicrobial peptides; PO, phenoloxidase.

host	immune challenge	tissues	stages	immunological dynamics	host phenotype	references
*Manduca sexta* (Lepidoptera)	none	gut	ecdysis at the larval to pupal transition	AMPs are prophylactically excreted into gut lumen during early metamorphosis		[20,21]
*Manduca sexta* (Lepidoptera)	*Photorhabdus luminescens*	fat body, haemocytes, cell-free haemolymph	pre-wandering and newly ecdysed larvae	cellular and humoral defences reduced upon entering metamorphosis	older larvae succumb faster to infection	[22]
*Manduca sexta* (Lepidoptera)	peptidoglycan	haemolymph	wandering larvae, pupae, and new adults	PO and AMP activity peak in larval stage, nadir in pupal stage		[23]
*Galleria mellonella* (Lepidoptera)	bacteria (*E. coli, M. luteus*) and fungi (*S. cerevisiae*)	haemolymph	larvae, pupae, adults	antimicrobial properties highest in pupae	immune challenge shortens development time, decreases pupal mass	[24]
*Galleria mellonella* (Lepidoptera)	none	haemolymph and cuticle	every day from last instar larva to new adult	PO activity lowest during late pupal stage		[25]
*Galleria mellonella* (Lepidoptera)	symbiotic (*E. mundii*) and pathogenic (*Serratia, Staphylococcus*) bacteria	gut	multiple stages of larval to pupal moult; adults	lysozyme and symbiont interaction important for excluding pathogens as pupae	pathogenic bacteria in pupal microbiota increased mortality hazard	[14,26]
*Bombyx mori* (Lepidoptera)	*S. aureus, E. coli* bacteria	gut	multiple stages of the larval to pupal moult	toll pathway AMPs highly expressed during ecdysis		[27]
*Bombyx mori* (Lepidoptera)	none	gut	feeding and wandering stage larvae; pupae	AMP expression increased just prior to pupation; changes in midgut morphology		[28]
*Danaus plexippus* (Lepidoptera)	*Ophyrocystis elektroscirrha* (protozoan)	haemolymph	larvae, adults	haemocyte count higher in larvae but PO activity higher in adults	individuals infected as larvae had shorter lifespans as adults	[29]
*Arctia plantaginis* (Lepidoptera)	none	whole body	multiple larval and pupal stages; adult	cold larval rearing temperatures increased larval and adult body melanization	larval body melanization trades off with antipredator coloration	[30]
*Drosophila melanogaster* (Diptera)	none	whole body	larvae, adults	AMPs differed in the strength of correlation between larval and adult expression	larval expression of the AMP drosomycin correlated with male offspring weight	[18]
*Drosophila melanogaster* (Diptera)	*Erwinia carotovora* (Ecc15)	gut, whole body	multiple larval and pupal stages; adult	Duox-controlled gene expression highly expressed in late larval and late pupal stages but declines during adulthood		[31]
*Anopheles gambiae* (Diptera)	*Enterobacter* or *E. coli*	haemolymph, whole body	larva and adult	haemocyte metrics differed between larvae and adults; generally higher in larvae	larval immune challenge increases adult susceptibility to *Plasmodium*	[17,32]
*Apis mellifera* (Hymenoptera)	lipopolysaccharide (LPS)	haemolymph	multiple larval, pupal, and adult stages	PO activity increased over development from larva to adult		[33]
*Apis mellifera* (Hymenoptera)	*E. coli*	haemolymph	larva, pupa, adult	AMP induction after bacterial exposure in pupae is much lower than other stages	pupae fail to clear bacteria and succumb to infection	[34]
*Carabus lefebvrei* (Coleoptera)	none	haemolymph	larvae, pupae, adult	haemocyte counts are much higher in pupae than in adults or larvae		[35]
*Nicrophorus vespilloides* (Coleoptera)	none	haemolymph	multiple larval stages, pupa, adult	haemocyte count lower but PO activity higher in pupae than in other stages		[36]

**Figure 1.**
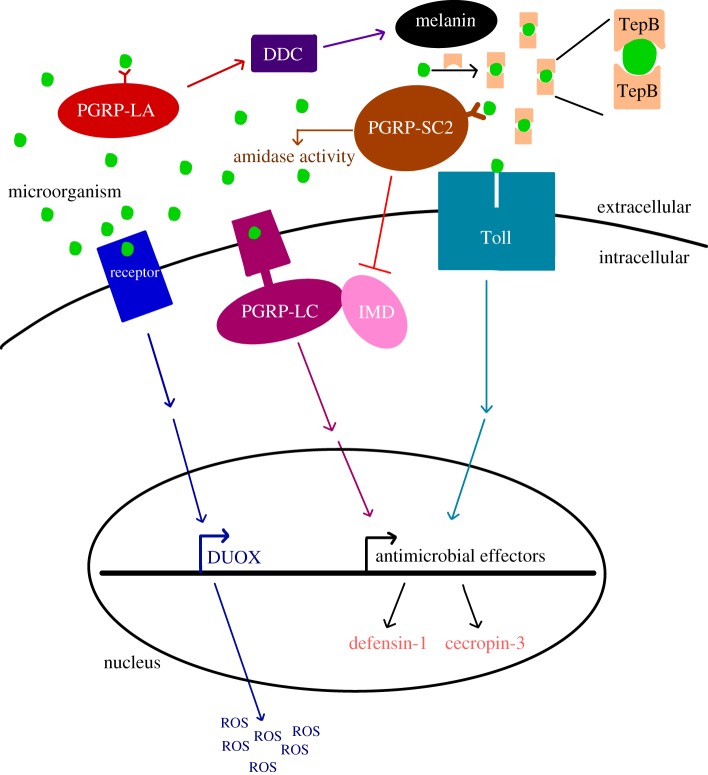
The proposed functional roles of *T. castaneum* immune genes quantified in this study. Peptidoglycan recognition proteins homologues (e.g. PGRP-LC and PGRP-LA) are thought to recognize parasites and stimulate signalling cascades that result in the production of antimicrobial effectors. The immune factors in this study are involved in the melanization pathway (DDC), production of reactive oxygen species (DUOX), opsonization by phagocytes (TepB) and degradation of microbial peptidoglycan via amidase activity (PGRP-SC2). The expression of antimicrobial peptides defensin-1 and cecropin-3 provide read-outs on the activation of Toll and IMD pathways. (Online version in colour.)

Despite the importance of complete metamorphosis for the outcome of host–parasite interactions, we know little about the legacy of larval infection on the immunological state of pupal and adult stages, particularly upon remodelling of the midgut. Most of what we do know (table 1) focuses on the response to immune challenge with bacteria. As both beneficial endosymbionts and virulent entomopathogens, bacteria are undoubtedly important selective factors on the adaptive decoupling of immune responses across life stages. Horizontally transmitted, relatively avirulent parasites like eugregarine protozoa are also ubiquitous among insect populations [27,37,38], and yet we know almost nothing about host immune responses to these parasites or their interaction with host metamorphosis. In this study, we compare the expression of immune genes in the guts and whole bodies of larval, pupal and adult flour beetles (*T. castaneum*) that were infected as larvae with a naturally occurring and common gregarine parasite that gets expelled with the recycled gut epithelia during metamorphosis. We chose to assay the expression of antimicrobial peptides (AMPs), recognition proteins and other immune effectors previously associated with the insect gut, metamorphosis and/or protozoan infection (figure 1). As flour beetles are holometabolic and have coevolved with a variety of parasites that afflict one or many stages [16], we predicted that different immune genes would show different strengths of pairwise correlations across beetle life stages, reflecting at least partial evolutionary decoupling of immune gene regulation. Even though gregarines are expelled from the gut during metamorphosis, adults can become re-infected, and we therefore also expected that larval infection would inform the expression of immune genes in pupae and adults in anticipation of adult re-exposure. We discuss the implications of our results for our understanding of the evolution of metamorphosis and innate immune systems.

## 2. Material and methods

### (a) Gregarine infections

Septate eugregarine protozoa are ubiquitous and generally avirulent inhabitants of insect midguts [14,27,39,40]. The strain of *Gregarina* parasites used in this study was originally derived from infected *T. castaneum* beetles collected at a feed store in Kentucky in June 2017 and subsequently maintained in a continuously infected colony. We have not observed any obvious disease-induced mortality or other symptoms of virulent infection with this parasite. This parasite is transmitted via the secretion of gametocysts from the infected insect gut. The gametocyst produces oocysts in the flour environment that are then ingested by the new host. Thus, the addition of beetle eggs to flour derived from a heavily infected colony is sufficient to reliably expose newly hatched larvae to the parasites [14,27,39,40]. Before the start of the experiment, we confirmed infection in the source colony by dissecting the guts of 15 mature larvae and staining with a 60% iodine saline solution to visualize gregarine parasites via light microscopy (25×). We found that 7/15 larvae had visible trophozoites in the midgut, although the infection rate is likely higher as the trophozoites are hard to see until almost ready to enter syzygy. None of the 15 pupae that we dissected showed signs of infection, agreeing with previous observations [14] that parasites are unlikely to survive in the pupal gut because the epithelia to which they are attached are destroyed.

### (b) Flour beetle rearing and sample collection

We set up 11 Petri dishes containing all-purpose white flour, to which we added 60 *T. castaneum* adults from the ‘Snave’ colony, originally collected from a Pennsylvania grain elevator in July 2013 and subsequently maintained in the laboratory [39]. Four days later, we sieved approximately 600 eggs from the breeding groups, mixed them together and distributed them randomly into one of two 0.5 l plastic containers, to which we added either 100 g of gregarine-positive flour from the heavily infected *T. castaneum* colony or 100 g of gregarine-free flour from a parasite-negative colony. Three weeks later, we pulled 50 pupae as well as 50 larvae with an approximate length of 4 mm from each treatment, and 25 newly eclosed, virgin adults from each treatment a week after that. For development assays, we collected 30 pre-pupae from each treatment and placed them in individual wells of a 96-well plate, monitoring them daily first for pupation and then for eclosion as new adults. All beetles were kept at 29°C in the same incubator in the dark except when handled.

All larval and adult individuals destined for gene expression studies were starved overnight prior to sample processing to eliminate non-colonized parasites and food in the midguts, although remnants remain in the hindgut. The beetles were then dipped in sterile water to remove excess flour immediately prior to sample collection. We dissected whole guts from all stages by making an incision in the abdomen and gently removing the gut with tweezers while the insect was immersed in 10 µl sterile insect saline. Guts were immediately placed in a 1.5 ml collection tube on dry ice. After collections were complete, guts were kept at −80°C. We originally treated a subset of guts with iodine as well to visualize parasites before freezing the guts, but after finding that iodine treatment severely affected gene expression, we eliminated these samples from subsequent analyses, leaving us with five to seven gut samples per exposure treatment per life stage. Whole individuals (8–10 per treatment/life stage) were placed in individual tubes, frozen and kept at −80°C.

### (c) Quantification of immune gene expression via RT-qPCR

We isolated gut RNA using the Qiagen All Prep Micro Kit and isolated whole body RNA with Qiagen All Prep and RNeasy kits. We synthesized cDNA with 0.5 µl RNA (whole body) or 4 µl RNA (gut) in a 5 µl or 10 µl reaction using the manufacturer-recommended protocol with SuperScript IV VILO master mix (ThermoFisher Scientific) and diluted the cDNA with 30–40 µl nuclease free water. We conducted RT-qPCR on the Biosystems Quantstudio 6 Flex machine using sybr green chemistry (PowerUp SYBR green master mix from Applied Biosystems, 500 nM primers (table 2), 10–50 ng cDNA). Thermal cycling conditions were 95°C for 2 min, followed by 40 cycles of 95°C (15 s), 55°C (10 s) and 60°C (1 min). All samples were run in duplicate or triplicate and the average ct value was used for subsequent analyses as long as technical replicates were within 1 ct.

**Table 2.** Primers used to assay immune gene expression in *T. castaneum.*

primer set	full name	function	forward oligo sequence	reverse oligo sequence	AT. (°C)
Def1	defensin-1	Toll/IMD AMP	TTTRYCGTTGCARTAKCCTCC	TCAARSTGAATCATGCCGCWTG	55
Cec3	cecropin-3	Toll AMP	AACATGARYACCAAACTTTT	CCAAYTTATMGGCTKTGGWG	55
PGRP-LA	peptidoglycan recognition protein LA	IMD recognition	TGCCACCTTAAACTTCTCTAAAC	GACTGCACCCTTTGCGAACAT	55
PGRP-LC	peptidoglycan recognition protein LC	IMD recognition	ACGAAGGCCGGGGATGGAAA	GTTGTTTGCAAGCCGTTATCTG	55
PGRP-SC2	peptidoglycan recognition protein SC2	IMD recognition	ACAGTTGGATGCKTTGAAACAGT	AACTSGTYCTGCTCCCTTG	55
DDC	dopa decarboxylase	melanin synthesis	AGAAGTCGTGATGCTKGACT	CTTGRATCACGCCGCC	55
Duox	dual oxidase	ROS synthesis	CGCAATTGATCGGCCACTTT	AGCTCCAAGGGATTTGGTCG	55
TEP-B	thioester-containing protein B	cellular recognition	AGGTTTCACCTCATCGCAGG	GTTGAAATTGTGGCGCTGGT	55
S18	ribosomal protein S18	ribosomal Protein	CGAAGAGGTCGAGAAAATCG	CGTGGTCTTGGTGTGTTGAC	55

We used RT-qPCR to quantify the expression of immune response-associated genes (figure 1) including *defensin-1, cecropin-3*, *dopa decarboxylase* (DDC), thioester-containing protein B (*TepB*), dual oxidase (*duox*) and the peptidoglycan recognition protein genes *pgrp-LA*, *pgrp-LC* and *pgrp-SC2*. Defensin-1 and cecropin-3 are AMPs that are thought to be activated by both the IMD and Toll pathways in *T. castaneum* and have orthologues that are upregulated during the bacterial oral challenge in *Bombyx mori* and *D. melanogaster* [41,42]. Pgrp-LA and pgrp-LC are transmembrane receptor proteins for the IMD pathway in *T. castaneum* and essential for its production of AMPs [43]. *PGRP-SC2* is the *T. castaneum* homologue of *pgrp-LB* in *D. melanogaster*, which downregulates the IMD pathway [44]. DDC is a precursor in the melanization pathway, which kills malaria parasites in the midgut of *Anopheles* mosquitoes [41,45]. TEPs are highly expressed in the crop and proventriculus in *D. melanogaster* [46]. Finally, Duox synthesizes reactive oxygen species (ROS) in gut epithelial cells, and RNAi knockdown of Duox has been shown to increase host susceptibility to oral bacterial infection in *D. melanogaster* [47]. We used *ribosomal protein S18* (*rps18*) as a reference gene for quantification of relative gene expression [48], as it has been shown to be stably expressed during infection [49] in *T. castaneum*. We confirmed its stability by comparing the ratios of mean S18 ct values to ng µl^−1^ cDNA among infected versus uninfected whole larvae (*F*_1,10_ = 3.09, *p* = 0.10), which was not significant.

### (d) Statistical analyses of gene expression

We calculated the *Δ*ct values for each gene for each individual sample by subtracting the mean ct value of the target gene from the reference gene mean ct value. Thus, the *Δ*ct value represents the relative expression value of the target gene on a log2 scale [48]. As our expression data were lognormally distributed, we retained the log2-transformed value for subsequent analyses. All statistical analyses were conducted in R (v. 3.5.2). To analyse the main effect of tissue on overall host gene expression, we conducted a MANOVA with our eight genes as dependent variables and tissue as the independent variable. To analyse the impact of each life stage, parasite exposure and their individual interactions within each tissue on gene expression, we used linear models (lm() function) of the form (target relative expression ∼ stage + exposure + stage * exposure). We adjusted the *p*-values with the Benjamini–Hochberg method to control for the false discovery rate [50]. We interpret a significant main effect of exposure as indicating the differential expression of a given gene in the same direction in multiple life stages, while a significant interaction effect would indicate that the magnitude or even direction of exposure-induced differential expression differs among stages. Finally, to analyse pairwise expression correlations between genes, we used the cor() function on gut and whole body as well as larval, pupal and adult data. To get differences in covariance relationships among these datasets, we subtracted the absolute value of one matrix from another and graphed the resulting differences using the lowerUpper (psych package) and ggcorrplot (ggcorrplot package) functions.

## 3. Results

### (a) The impact of gregarine infection on pupal development

The majority of individuals from both treatment groups took 6 days to develop from newly ecdysed pupae to newly eclosed adults and thus the distribution of development times was underdispersed (dispersion parameter = 0.05). Nevertheless, individuals exposed to gregarine parasites as larvae developed significantly faster than those who were not exposed (quasi-Poisson GLM, *t* = 2.05, *p* = 0.046), although the effect size was less than 1 day among treatments.

### (b) Immune gene expression differs by tissue

**Figure 2.**
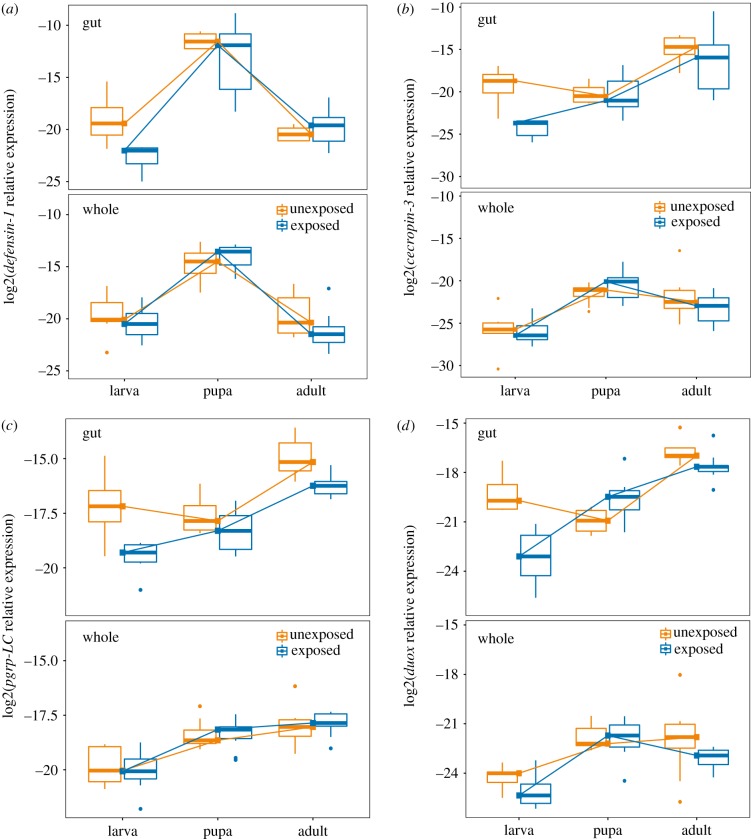
The influence of tissue type and gregarine parasite exposure on immune gene expression across developmental stages of the flour beetle *T. castaneum*. The expression of the antimicrobial peptides *defensin-1* (*a*), and *cecropin-3* (*b*)*,* the recognition protein *pgrp-LC* (*c*) and the reactive oxygen species generator *duox* (*d*) were assayed in extracted guts (top row of each panel) or whole bodies (bottom row) from larvae, pupae or adults that were either exposed to gregarine parasites as larvae (blue; right boxes) or not (orange; left boxes). The expression of each gene relative to the reference gene *RP18s* is represented on a log2 scale. Lines have been added to visualize the developmental trajectory of median gene expression. (Online version in colour.)

The overall effect of tissue (gut versus whole body) on gene expression was highly significant (MANOVA, *F*_1,89_ = 31.34, *p* < 2 × 10^−16^). Models for individual genes revealed that the gene *ddc* had, on average, 5.7-fold higher expression in the whole body than in the gut (*F*_1,89_ = 27.7, *p* < 1 × 10^−6^). Genes that showed significant upregulation in the gut relative to the whole body, on the other hand, include *pgrp-LC* (fold change = 2.6, *F*_1,89_ = 21.4, *p* < 2 × 10^−5^), *duox* (fold change = 11.47, *F*_1,89_ = 66.2, *p* < 1 × 10^−11^) and *cecropin-3* (fold change = 14.8, *F*_1,89_ = 31.4, *p* < 1 × 10^−6^). There was no significant tissue-driven difference in expression for the genes *pgrp-LA, defensin-1, pgrp-SC2* or *tepB* (*p* > 0.05). Figure 2 illustrates the relative expression of four genes among tissues (top row in each panel = gut expression, bottom row = whole body expression).

**Figure 3.**
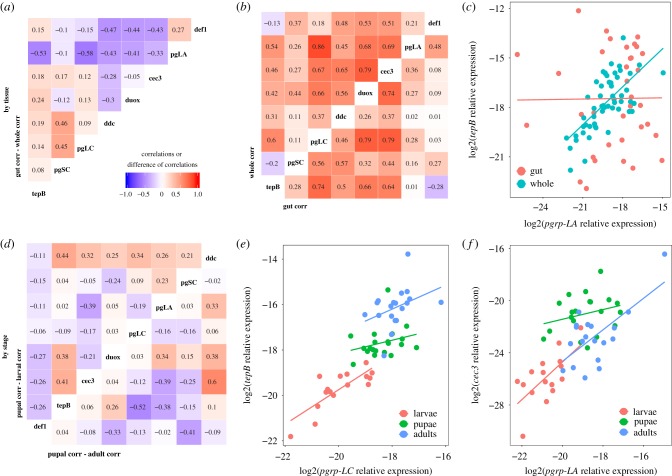
Gene expression correlations suggest partial decoupling of immune genes between tissues and among life stages. The pairwise Pearson correlation values of whole-body gene expression were subtracted from those of gut-only pairwise correlations to get the difference in correlation strength (*a*). Large positive values indicate a stronger relationship in the gut, while large negative values indicate stronger correlations in the whole body. The underlying correlations are visualized in (*b*) for whole body (top left) or gut only (bottom right); colours and numbers indicate the Pearson correlation coefficient. The breakdown of the correlation of *pgrp-LA* and *tepB* expression (log2 scale, relative to reference gene) in the gut relative to the whole body (*c*) illustrates decoupling among tissues. There was also decoupling by life stage, as illustrated by the relative magnitudes of the correlation coefficients for pupae against larvae (*d*, top left) and pupae against adults (*d*, bottom right). Stage-specific pairwise comparisons of *pgrp-LC* versus *tepB* expression (*e*) and *pgrp-LA* versus *cecropin-3* expression (*f*) illustrate different examples of differences in coefficients among stages. (Online version in colour.)

Does the co-regulation of immune gene expression differ between tissues? Previous work on *T. castaneum* and other model insects like *D. melanogaster* have proposed that several of our genes are likely to be under the control of common pathways like IMD and Toll (e.g. [51]), resulting in expression patterns that covary among co-regulated genes. In our data, all genes showed a moderate to high correlation of expression with at least one other assayed gene (figure 3*b*), but these relationships were not always consistent among tissues (figure 3*a*). For example, *pgrp-LA* and *tepB* were tightly correlated at the whole body level but show no relationship in the gut (figure 3*c*).

### (c) The effect of developmental stage and parasite exposure on immune gene expression

To analyse the impact of developmental stage, larval gregarine exposure and their interaction on immune gene expression (figure 2), we performed linear modelling on each gene. We analysed gut and whole body datasets separately because of the complex tissue-specific genic interactions described above. In the whole body, there was no significant effect of gregarine exposure on gene expression (table 3), but expression differed broadly by life stage. Most genes showed higher expression in pupae and adults relative to larvae (electronic supplementary material, table S1 ‘whole body’). *Pgrp-LC*, *pgrp-LA* and *tepB* increased in each subsequent life stage, while *defensin-1* (figure 2*a*) and to a lesser extent *cecropin-3, duox* and *ddc* peaked in the pupal stage. Only *pgrp-SC2* expression showed no significant effect of stage.

**Table 3.** Summary of statistical results for the impact of stage, larval parasite exposure or their interaction on immune gene expression in the gut and whole body. Full statistical tables for each gene are available in electronic supplementary material, table S1. The expression of each gene was fit with the model: expression ∼ stage × exposure using the lm() function in R, where stage has three levels (larva, pupa, adult) and parasite exposure has two levels (exposed, unexposed). *p*-values were adjusted for false discovery rate using the Benjamini–Hochberg method, and asterisks indicate the level of significance for at least one level of factor or interaction, relative to unexposed larvae: *p_adj_ < 0.05, **p_adj_ < 0.01, ***p_adj_ < 0.001. ‘—’ indicates lack of statistical significance.

	gut			whole body		
gene	stage	exposure	stage*exposure	stage	exposure	stage*exposure
*defensin-1*	***	—	—	***	—	—
*duox*	*	***	***	***	—	—
*tepB*	***	—	—	***	—	—
*cecropin-3*	—	*	—	***	—	—
*ddc*	—	—	—	**	—	—
*pgrp-LC*	*	**	—	***	—	—
*pgrp-LA*	—	—	—	***	—	—
*pgrp-SC2*	—	—	—	—	—	—

The expression of immune genes in the gut was more diverse in the response to stage and exposure (table 3). Larval exposure to gregarines resulted in the overall downregulation of *cecropin-3* (figure 2*b*), *pgrp-LC* (figure 2*c*) and *duox* (figure 2*d*) that persisted into the pupal and adult stages. The expression of *duox* further depended on the interaction of exposure and life stage (electronic supplementary material, table S1), as the expression was suppressed in exposed larvae but upregulated in the guts of pupae that were previously exposed (figure 2*b*). Only *defensin-1* was more highly expressed in pupae than in larvae and adults (figure 2*a*), but *pgrp-LC, duox* and *tepB* were significantly more highly expressed in adults relative to larvae (e.g. figure 2*c,d*). No gene was most highly expressed in larvae than in other life stages.

In whole organisms, the strength of pairwise gene expression correlations differed among life stages (figure 3*d*). For example, *pgrp-LC* and *tepB* expression was tightly and steeply correlated in larvae but less so in pupae and adults (figure 3*e*), while the strong positive relationship observed between *pgrp-LA* and *cecropin-3* in larvae and adults broke down in pupae (figure 3*f*).

## 4. Discussion

Our data suggest that larval exposure to a relatively benign protozoan parasite can leave an imprint on gut immune system gene expression that persists into metamorphosis. Our study also reflects a dynamic change in the immunological profile of the insects as they mature through metamorphosis into adulthood and demonstrates the decoupling of immune gene regulation among tissues as well as across different life stages. As we know little about the insect immune response to eugregarines despite their ubiquity and diversity, and moreover the immunological dynamics of metamorphosis are still poorly described for most insects, this study provides a unique window into the integration of parasites with the life history of holometabolous insects. The persistent downregulation of several important immune recognition and effector genes beginning in the gut of infected larvae also raises the possibility of trade-offs with resistance to the bacterial infection that could haunt the host in later life stages.

The downregulation of AMPs observed in infected larval guts in our study may reflect parasitic manipulation of IMD and Toll pathways, but it could also hint at the polarization of the immune response toward defences aimed at eukaryotic parasites at the expense of antibacterial defences. Evidence from the Egyptian cotton leafworm (*Spodoptera littoralis*), for example, suggests that antibacterial activity trades off with cellular immune function and cuticular melanization in larvae [52]. While the insect immune response to gregarines has not been well described prior to this study, we do have evidence that gregarine infection can impact concurrent or subsequent infections. For example, larval *T. confusum* infection with the gregarine parasite *Gregarina minuta* primed the resulting adults to better resist re-infection [14] although the impact of gregarines across generations was less beneficial to their flour beetle hosts; gregarine-infected *T. confusum* females produced offspring that were more susceptible to infection with the virulent bacterial entomopathogen *Bacillus thuringiensis* [39]. Gregarines do not always facilitate entomopathogens, however, as cockroaches (*Blattella germanica*) were less competent hosts for parasitic nematodes if they were first infected with gregarines [53]. While the polarization of helper-T cell responses [54], for example, is well characterized in mammals, we still have not delineated analogous mechanisms that contribute to functional trade-offs among arms of the immune system in insects. Gregarine infection may represent an underappreciated route for exploring the costs of maintaining multiple immunological fronts in invertebrate immune systems.

Should we expect decoupling of immune gene regulation across discrete life stages in flour beetles? Larval, pupal and adult flour beetles all live in the same milled grain substrate and therefore experience similar environmental challenges [55], including exposure to gregarine parasites, suggesting that selection for stage-specific immune system optimization may not be as extreme as in insects that experience completely different ecological conditions over ontogeny. However, there are still fine-scale spatial and behavioural differences among the flour beetle life stages that could bias relative rates of parasite exposure. Larvae tend to burrow down into the flour column and are renowned for their tendency to cannibalize multiple life stages [56], making the transmission of parasites an occupational hazard. Adults, who generally only cannibalize eggs and then only under high-density conditions, tend to congregate at the top of the column where they can find mates or achieve dispersal. Pupae also congregate at the top of the column, cannot feed and lack robust behavioural defences, making them easy targets for both predators and parasites or parasitoids that can penetrate the cuticle. Finally, mating is a well-known test of adult-specific immunological competence, as exposure to sexually transmitted diseases and the resource-intensive costs of producing offspring can tax host defences [33].

Thus, we might still expect selection to favour adaptive decoupling of immunological architecture in this system, and our data suggest that immune gene co-regulation does become at least partially decoupled across life stages. For example, the recognition gene *pgrp-LA* and the antimicrobial peptide gene *cecropin-3* are tightly positively correlated in larvae and adults, but the correlation is completely lost in pupae (figure 3*f*). Co-regulation was also decoupled across tissues, as *pgrp-LA* and *tep-B* are strongly positively correlated at the whole body level but completely uncorrelated in the gut (figure 3*c*). This agrees in part with previous work on the ontogenic [19] and tissue-specific [31] decoupling of AMP expression in *D. melanogaster*, and our work additionally suggests that there might be an interaction between stage and tissue. This makes sense from an evolutionary perspective, as some parasites to which the flour beetle is exposed mainly inhabit the gut (e.g. eugregarines and the microbiota), while others inhabit the haemolymph or fat body (e.g. microsporidia or coccidia [16,35]), requiring different responses and regulatory mechanisms in different tissues. Thus, we advise caution when inferring the contributions of particular immune pathways or regulatory elements to differentially expressed immune genes, as many canonical immune pathways have been described from studies of whole adult insects. This caveat extends to the study of immunological imprinting from early-life infection, as the signal of gregarine infection on immunity across ontogeny was lost at the whole-organism level.

Most of our immune genes were significantly differentially expressed in different life stages at the whole organism level independent of gregarine infection, and a subset was also significant in the gut for the main effect of life stage. Our observation of monotonically increasing recognition and effector gene expression over ontogeny is consistent with a few examples from table 1 (e.g. PO activity in *Apis mellifera* [57]) but at odds with others that demonstrate peak responses during larval [18,20] or pupal [58] stages. Only *defensin-1*, for an antimicrobial peptide that tends to be highly expressed in both the presence and absence of infection in flour beetles [59], peaked in expression during the pupal stage (figure 2*a*), consistent with observations in lepidopteran hosts of high antimicrobial peptide expression against opportunistic infections by microbes escaping the gut lumen during metamorphosis [15,60]. We note that we collected the larvae and pupae at the same time for this experiment, and while the difference in their development times may be down to the length of the egg laying period, it is also possible that the pupae were higher quality individuals able to develop faster. However, in this case, we would expect most genes to show non-monotonic expression over development, so differences in quality are unlikely to be a major factor here. Future work using stage- and tissue-specific functional genetics approaches could help to clarify the relative contributions of canonical and non-canonical immune pathways and host quality to the generation of antimicrobial effectors across tissues and life stages.

Moving forward, how can we assess the role of infection and immunity in the evolution of metamorphosis, and conversely the role of metamorphosis in immune system evolution? First, it would be interesting to leverage the overlap in stage-structured ecological niches among holometabolous and hemimetabolous insects such as mosquitoes and damselflies or milkweed bugs and milkweed beetles to characterize, for example, patterns of parasite prevalence or immune function as a function of environment, stage and developmental mode. With the maturation of the i5 k project [21] and related efforts to sequence and annotate insect genomes, comparative analyses of immune gene architecture or stage-structured transcriptional dynamics among species could help to disentangle the effects of phylogeny from ecology and ontogeny on immune system evolution. In addition, better characterization of the natural enemies of insects could complement current descriptions of immunological dynamics in model insects against laboratory-amenable bacteria. For example, the relative rates of exposure and susceptibility metrics could differ among life stages, as could the density distribution of parasites among infected insects. Many parasites are aggregated among hosts (including gregarines, although we could not quantify parasite load distribution in this study). If most hosts have only a few parasites while a few hosts have many parasites [22], parasite aggregation could induce heterogeneity in the impact of infection on host demography through mortality or developmental effects. Connecting empirical patterns of infection and immunity across ontogeny with mathematical models of age- and stage-structured immune system evolution [2,7,23] could provide a unifying framework for understanding patterns of immunological variation in nature.

## Supplementary Material

Table S1

## Supplementary Material

Data

## Data accessibility

The data are provided as electronic supplementary material.
